# Comparative Population Genetics of *Rutilus rutilus* and *Perca fluviatilis* in Lithuanian Freshwater Ecosystems

**DOI:** 10.3390/ani16162540

**Published:** 2026-08-14

**Authors:** Ieva Ignatavičienė, Adomas Ragauskas, Adrijana Bencevičiūtė, Vytautas Rakauskas, Dalius Butkauskas

**Affiliations:** State Scientific Research Institute Nature Research Centre, Akademijos Str. 2, 08412 Vilnius, Lithuania; adomas.ragauskas@gamtc.lt (A.R.); adrijana.benceviciute@gmail.com (A.B.); vytautas.rakauskas@gamtc.lt (V.R.); dalius.butkauskas@gamtc.lt (D.B.)

**Keywords:** mitochondrial DNA, gene flow, dispersal, sympatric species, population structure, drainage basins, postglacial history, Baltic region

## Abstract

Understanding how co-occurring freshwater species respond to the same hydrological landscape is important for interpreting genetic diversity and population connectivity. We compared the population genetic structure of two widespread freshwater fishes, *Rutilus rutilus* (Linnaeus, 1758) and *Perca fluviatilis* Linnaeus, 1758, across major Lithuanian river basins using mitochondrial ATP6 and D-loop sequences. Both species exhibited high haplotype diversity but differed markedly in genetic structure. *R. rutilus* showed weak differentiation, widespread haplotype sharing among basins, and demographic signals compatible with population expansion. By contrast, *P. fluviatilis* exhibited stronger, more spatially heterogeneous structuring, with less consistent demographic signals and more localized differentiation. Contrary to expectations, genetic variation was not consistently associated with major drainage-basin boundaries in either species. Instead, the observed patterns suggest that species-specific ecological traits, particularly differences in dispersal capacity and habitat use, may play a greater role in shaping population structure than contemporary hydrological boundaries alone. These findings demonstrate that co-occurring freshwater fishes can exhibit contrasting patterns of genetic diversity even within shared hydrological systems. Although nuclear or genome-wide markers would strengthen these mitochondrial inferences, the study highlights the value of comparative approaches for understanding population connectivity and informing freshwater biodiversity conservation and management.

## Simple Summary

Freshwater fish populations are increasingly affected by habitat fragmentation, environmental change, and human activities. Understanding how fish populations remain connected across rivers and lakes is important for biodiversity conservation and sustainable fisheries management. In this study, we compared two common freshwater fish species, the common roach and the Eurasian perch, collected from major river basins in Lithuania. Although both species live in the same freshwater environments, they showed different patterns of genetic variation. The common roach displayed greater connectivity among populations, whereas the Eurasian perch showed stronger differences among locations. These results suggest that species can respond differently to the same environmental conditions because of differences in their ecology and movement behaviour. Our findings highlight the importance of considering species-specific characteristics when assessing freshwater biodiversity and developing conservation and management strategies for aquatic ecosystems.

## 1. Introduction

Freshwater ecosystems are among the most fragmented, with the genetic diversity of the organisms living in them shaped by both hydrological connectivity and historical landscape configuration. In Northern Europe, repeated Pleistocene glaciations reshaped drainage networks and aquatic habitats, leaving long-lasting traces in the genetic structure of many freshwater taxa [1]. Therefore, the current structure of genetic diversity reflects both historical processes and current environmental conditions [1,2,3], and disentangling their relative contributions remains one of the main goals of freshwater population genetics.

One way to address this problem is to compare species that occur together in the same hydrological landscape but differ in traits that may influence gene flow. Co-occurring fishes often differ in life cycle, distribution, habitat use, dispersal, and site fidelity, all of which can shape population genetic structure [4,5,6]. Species with greater dispersal may maintain higher connectivity across hydrological boundaries, whereas stronger site fidelity and more restricted movement may favour greater population differentiation. Because co-occurring species experience the same broad environmental and hydrological history, comparing their genetic structure can help separate the effects of shared landscape history from those of species-specific ecology, and test whether hydrological barriers leave concordant genetic signatures among coexisting taxa or whether ecological differences override that shared history [7,8,9].

We apply this comparative framework to two widespread but ecologically contrasting fishes, the common roach (*Rutilus rutilus* (Linnaeus, 1758)) and the Eurasian perch (*Perca fluviatilis* Linnaeus, 1758), which co-occur across the major river basins of Lithuania. *R. rutilus* is a highly adaptable cyprinid that occupies diverse freshwater habitats and undertakes seasonal movements between feeding and spawning areas [10,11], potentially promoting gene flow among connected water bodies. *P. fluviatilis*, by contrast, shows stronger site fidelity and generally more limited movement, with many populations completing their life cycle within a single water body [12,13]. Consistent with these ecological differences, earlier work has reported comparatively weak genetic structuring in *R. rutilus* and stronger, more spatially heterogeneous differentiation in *P. fluviatilis* [7,9,14,15]. Because the two species co-occur in the same lowland water bodies and share a broadly similar regional glacial and hydrological setting yet differ markedly in dispersal and site fidelity, they provide a natural comparison for assessing the role of species-specific ecology in genetic structure.

Lithuania is geographically located in the drainage basin of the Baltic Sea and encompasses four main basins—the Nemunas, Lielupe, Venta, and Daugava—that ultimately reach the Baltic but remain largely hydrologically independent, with few natural interconnections among them. These drainage divides constitute discrete, well-defined hydrological units within a comparatively small geographic area, so that the same set of potential barriers can be evaluated simultaneously for both species using the same sampling design and molecular markers. This regional focus does not capture species-wide phylogeography, but it provides the controlled design needed to isolate the contribution of contemporary hydrological boundaries to genetic differentiation.

Although *R. rutilus* and *P. fluviatilis* have each been studied extensively as single species [7,9,10], direct comparisons of the two within the same landscape using the same sampling design and molecular markers remain rare, particularly for mitochondrial data from the Baltic region. This gap makes it difficult to separate the relative roles of species-specific ecology and shared hydrology, which are otherwise inferred across studies that differ in sampling design, markers, and geographic coverage. The distinction also has practical consequences: intraspecific genetic diversity underpins adaptive potential and resilience to environmental change [16], and management units defined by hydrology alone may not capture biologically meaningful connectivity for every species. To address this gap, we characterised mitochondrial DNA (mtDNA) variation using the ATP6 gene and the control region (D-loop), markers widely used in freshwater population genetics and phylogeography for their sensitivity to demographic and historical processes [1,17,18,19,20]. Because mtDNA constitutes a single, maternally inherited locus, we interpret the results as reflecting only part of each species’ evolutionary history and treat demographic inferences as signals rather than definitive reconstructions.

Accordingly, we compared the population genetic structure of *R. rutilus* and *P. fluviatilis* across the major Lithuanian basins to evaluate the relative importance of hydrological boundaries versus species-specific ecology and, given the region’s postglacial recolonisation history [1,20], to characterise the demographic signals retained in each species. Using the same two mitochondrial markers (ATP6 and D-loop) and a common analytical framework, we tested two complementary hypotheses: (i) if structure is driven by shared hydrology, drainage-basin boundaries should be associated with genetic differentiation, with a significant among-basin component, in both species; and (ii) if species-specific ecology predominates, the two species should differ in the extent and spatial pattern of differentiation, despite sharing the same hydrological landscape and analytical framework. Disentangling these alternatives will help clarify how contemporary hydrological boundaries and species-specific ecological traits together shape genetic diversity in Lithuanian freshwater ecosystems [18,19].

## 2. Materials and Methods

### 2.1. Sample Collection

**Figure 1:**
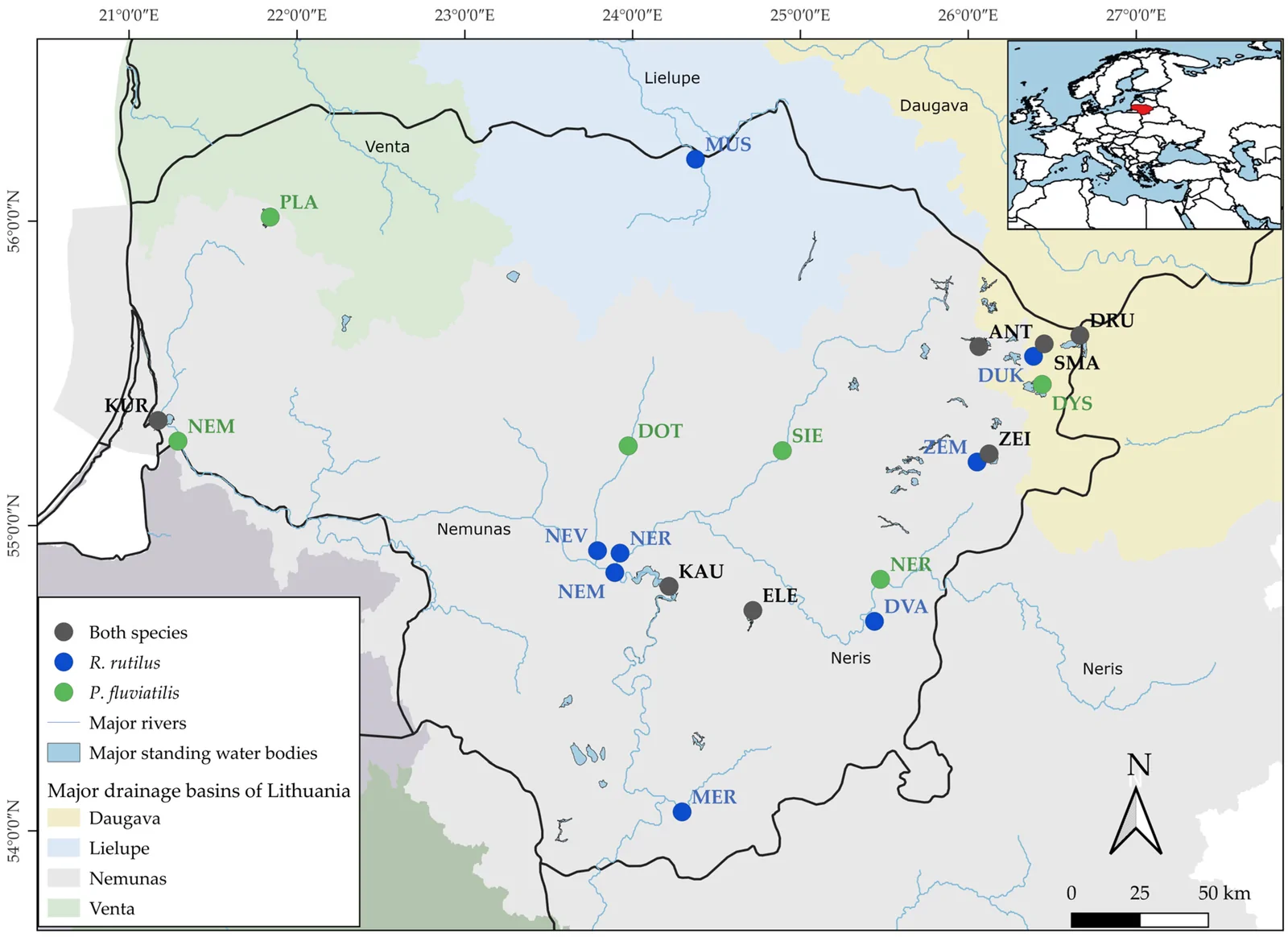
Sampling sites of *R. rutilus* and *P. fluviatilis* in Lithuania.

A total of 209 individuals of *R. rutilus* and 191 individuals of *P. fluviatilis* were collected from freshwater systems across Lithuania, including rivers, lakes, reservoirs, and the Curonian Lagoon (Figure 1; Table S1). Sampling was conducted between 2009 and 2025 using standardized fisheries methods described in previous studies [18,19,20,21]. In lakes and reservoirs, fish were collected using multimesh benthic gillnets (40 m length, 3 m height) with mesh sizes ranging from 14 to 60 mm. Riverine sites were sampled using battery-powered electrofishing, conducted either by wading or from a boat for 10 min intervals at depths of 0.5–2.0 m. All sampling was performed during late summer to early autumn to minimize seasonal variation in population structure. Sampling was conducted under permits issued by the Lithuanian Environmental Protection Agency. Species identification followed Kottelat and Freyhof [22], and taxonomy was consistent with FishBase [23].

### 2.2. DNA Extraction, PCR and Sequencing

DNA extraction, PCR amplification, and sequencing procedures followed established protocols [18,19,20,21,24,25,26], with minor modifications. Genomic DNA was extracted from frozen muscle tissue in *R. rutilus* and from frozen muscle and/or fin tissue in *P. fluviatilis*. For recently collected *P. fluviatilis* samples (2025), a combination of muscle and caudal fin tissue was used. Across the entire sampling period, tissue samples were stored frozen (−20 °C) after collection, and DNA extraction and sequencing were carried out within several months of collection for each sample, minimising any effect of storage duration on DNA quality. For amplification of the *R. rutilus* mtDNA D-loop region, a newly designed forward primer (Rut_1F: 5′-TTCTGATGGTCGCGTATATGA-3′) was used to improve amplification success, replacing a previously applied primer (Rut_2F). The reverse primer (Rut_2R: 5′-AGGTCAGGACCATGCCTTTA-3′) remained unchanged from previous studies [18,21]. The same D-loop region was targeted, although a slightly shorter fragment was amplified. The ATP6 region was amplified using previously published primers [18,21], and primer sequences are provided in those references. For *P. fluviatilis*, previously published primer sets for both ATP6 and D-loop regions were used without modification [19,27].

PCR reactions were carried out in 25 µL volumes containing 12.5 µL of 2× Taq Master Mix (Vazyme, Nanjing, China), 5.5 µL nuclease-free water, 1 µL of each primer, and 5 µL of genomic DNA (50–100 ng/µL). Thermal cycling conditions consisted of an initial denaturation at 95 °C for 2 min, followed by 35 cycles of 94 °C for 15 s, annealing at 58 °C (*R. rutilus* D-loop) or 52 °C (all other reactions) for 15 s, and extension at 72 °C for 30 s, with a final extension at 72 °C for 5 min. PCR products were purified using *Exo*I and FastAP enzymes (Thermo Fisher Scientific Baltics, Vilnius, Lithuania) and sequenced using the BigDye^®^ Terminator v3.1 Cycle Sequencing Kit on an ABI 3500 Genetic Analyzer (Applied Biosystems, Waltham, MA, USA). Singleton haplotypes were confirmed by repeated sequencing. Due to differences in sequencing performance among primers, slightly shorter but higher-quality fragments were used for downstream analyses. Final aligned fragment lengths were approximately 454 bp (ATP6) and 368 bp (D-loop) for *R. rutilus*, and 627 bp (ATP6) and ~324 bp (D-loop) for *P. fluviatilis*. To enable broader phylogeographic comparison, publicly available sequences from GenBank were included where combined ATP6–D-loop haplotypes from the same individuals were available.

### 2.3. Data Analysis

Sequences were aligned in MEGA X [28] for *R. rutilus* and MEGA 11 [29] for *P. fluviatilis*, using the MUSCLE [30] and ClustalW [31] algorithms, respectively, and all alignments were inspected and verified manually. The retained indels were short and unambiguously positioned within the D-loop, whereas ambiguous heteroplasmic length variants (10–20 bp insertions in the *P. fluviatilis* D-loop) were excluded. Because the aligned sequences showed low divergence and contained only short, unambiguous indels, the choice of software version and alignment algorithm is not expected to have affected haplotype identification or subsequent analyses. Standard indices of genetic diversity, including the number of polymorphic sites (S), number of haplotypes (h), haplotype diversity (Hd), nucleotide diversity (π), and mean number of pairwise differences (k), were calculated in DnaSP v6.0 [32] from the concatenated ATP6–D-loop sequences.

Median-joining haplotype networks were constructed using NETWORK v10.2.0.0 [33], with an additional maximum parsimony filtering step [34,35] to reduce network complexity. Haplotype clusters (A–D) were identified based on network topology for descriptive purposes only and do not represent phylogenetically supported lineages.

Phylogenetic relationships were reconstructed using the Maximum Likelihood (ML) method implemented in MEGA X [28] for *R. rutilus* and MEGA 11 [29] for *P. fluviatilis*. The best-fitting nucleotide substitution model was selected separately for each species based on the Bayesian Information Criterion [36]. The HKY+G model was applied for *R. rutilus*, whereas the HKY+I model was used for *P. fluviatilis* [37]. Due to the lack of available mitochondrial sequences of closely related taxa in GenBank, *Scardinius erythrophthalmus* (Linnaeus, 1758) was selected as the most suitable available outgroup for *R. rutilus*, and *Perca flavescens* (Mitchill, 1814) was used as an outgroup for *P. fluviatilis*. Branch support was assessed using bootstrap analysis with 1000 replicates [38].

Genetic differentiation among populations was assessed using pairwise F_ST_ values calculated in Arlequin v3.5.2.2 [39] based on concatenated ATP6–D-loop sequences. Negative F_ST_ values were treated as zero. Statistical significance (*p* < 0.05) was evaluated using 10,000 permutations. To test directly whether genetic variation is associated with drainage-basin structure, we performed a hierarchical analysis of molecular variance (AMOVA) in Arlequin v3.5.2.2 using concatenated ATP6–D-loop sequences, with sampling sites assigned a priori to their respective drainage basins. Variation was partitioned among basins (F_CT_), among populations within basins (F_SC_), and within populations (F_ST_), using pairwise differences as the molecular distance. The significance of each component was assessed with 10,000 permutations. Because basin coverage differed between species according to sampling, *R. rutilus* populations were grouped into three basins (Nemunas, Daugava, and Lielupe, the last represented by a single site), whereas *P. fluviatilis* populations spanned two (Nemunas and Daugava).

Demographic history was inferred using multiple complementary approaches. Neutrality tests, including Tajima’s D [40] and Fu’s Fs [41], were performed in Arlequin with 10,000 permutations. Mismatch distribution analyses were performed in Arlequin by fitting sudden demographic and spatial expansion models. Goodness-of-fit was assessed using the sum of squared deviations (SSD) and Harpending’s raggedness index, with statistical significance evaluated based on 10,000 coalescent simulations [42]. Mismatch distributions were visualized using DnaSP.

Historical demographic dynamics were inferred using Bayesian Skyline Plot (BSP) analysis in BEAST v2.7 [43]. Input files were prepared in BEAUti v2.7 applying the HKY+G substitution model (four gamma rate categories, estimated base frequencies). A strict molecular clock was calibrated using a per-lineage substitution rate of 1 × 10^−8^ substitutions/site/year, corresponding to a pairwise divergence rate of 2% per million years commonly assumed for fish mtDNA [25,44], and a Coalescent Bayesian Skyline tree prior (five groups) was used. MCMC chain lengths were increased incrementally for each species until all key parameters reached effective sample sizes (ESS) > 200, as assessed using Tracer v1.7.1 [45]. This required 100 million generations for *R. rutilus* and 10 million generations for *P. fluviatilis*. Chains were sampled every 1000 steps, with the first 10% discarded as burn-in.

## 3. Results

### 3.1. Sequence Characteristics and Polymorphism

The concatenated ATP6–D-loop sequences revealed genetic variation in both studied species, with most nucleotide positions being invariant. In *R. rutilus*, a total of 209 individuals from 15 sampling sites were analyzed. Among Lithuanian samples, most nucleotide positions were invariant (95.38% of alignment positions), with 37 polymorphic sites identified, including two indel events. Incorporation of additional sequences from GenBank increased the total number of polymorphic sites to 60, reflecting the inclusion of more divergent haplotypes. All indels were located within the D-loop region.

In *P. fluviatilis*, 191 individuals from 13 sampling sites were analyzed. Similarly, most nucleotide positions were invariant (96.52% of alignment positions), with 33 polymorphic sites identified, including four indels. After inclusion of GenBank sequences, the number of polymorphic sites increased to 39. As in *R. rutilus*, all indels were confined to the D-loop region.

Overall, *R. rutilus* showed a slightly higher number of polymorphic sites compared to *P. fluviatilis*. Several individuals of *P. fluviatilis* displayed heteroplasmic length variants (10–20 bp insertions) in the D-loop region. These variants were excluded from subsequent analyses as they could not be reliably aligned across all samples, to ensure alignment consistency and avoid potential biases in diversity estimates and haplotype network construction.

### 3.2. Population Genetics and Structure

Genetic diversity indices varied among populations of both species (Table 1). In *R. rutilus*, haplotype diversity (*Hd*) ranged from 0 (ZEI) to 0.958 (SMA), with an overall value of 0.760 ± 0.031. Nucleotide diversity (*π*) ranged from 0 to 0.0025, with a mean of 0.0017 ± 0.0001. A total of 37 polymorphic sites and 49 haplotypes were identified among 209 sequences, with an average number of nucleotide differences (*k*) of 1.402. Most populations exhibited relatively low nucleotide diversity, although higher values were observed in several populations, including SMA, NEV, and KAU.

In *P. fluviatilis*, haplotype diversity ranged from 0.105 (PLA) to 0.933 (NER), with an overall value of 0.892 ± 0.012. Nucleotide diversity ranged from 0.0002 to 0.0046, with a mean of 0.0032 ± 0.0001. A total of 33 polymorphic sites and 46 haplotypes were detected among 191 sequences, with an average number of nucleotide differences (*k*) of 3.619. Several populations, including DOT, SMA, and ANT, exhibited higher nucleotide diversity values compared to other sampling sites. Overall, *P. fluviatilis* showed higher haplotype diversity, nucleotide diversity, and mean number of nucleotide differences than *R. rutilus*.

**Figure 2:**
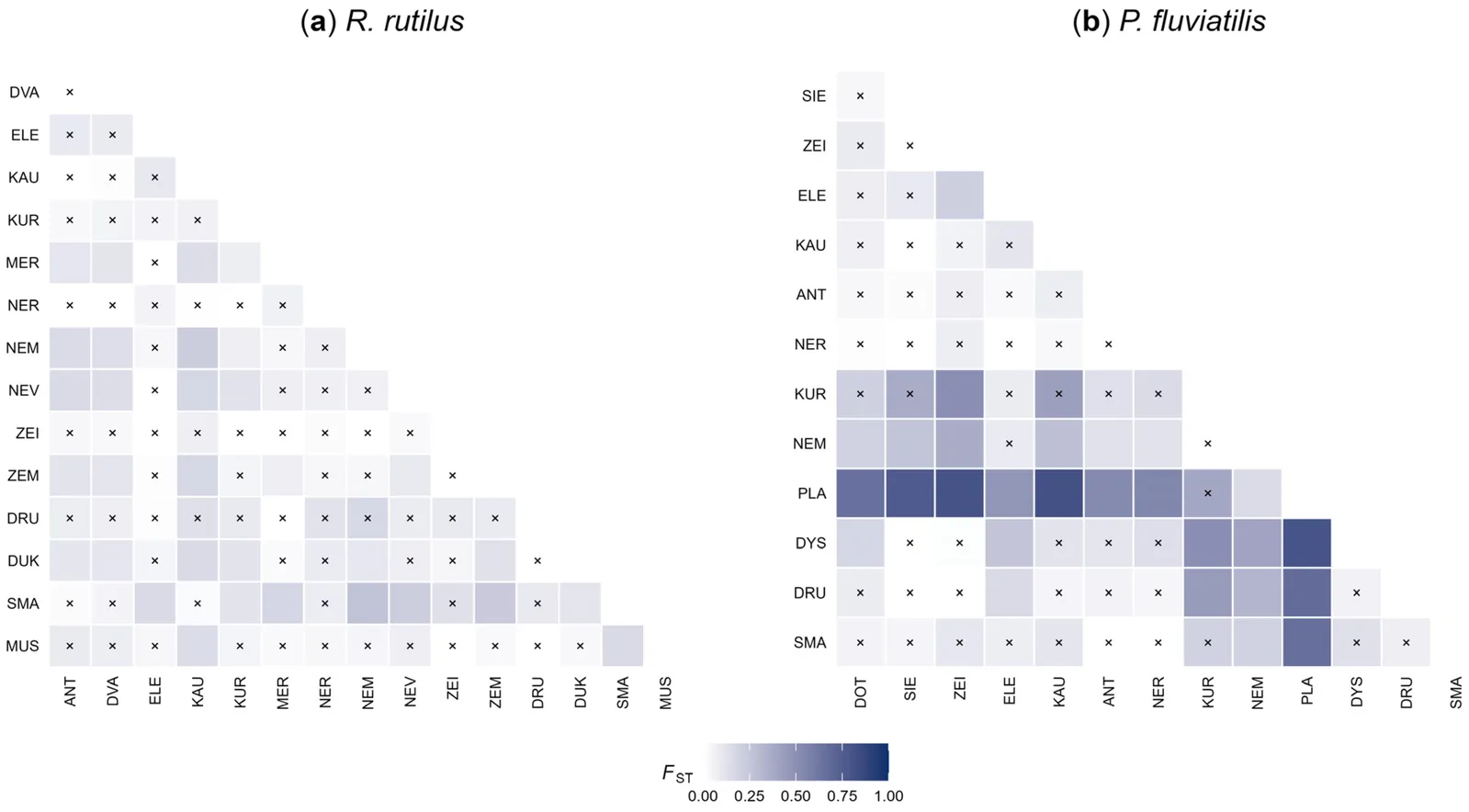
Matrix showing pairwise differentiation estimates (F_ST_) among: (**a**) 15 samples of *R. rutilus*; (**b**) 13 samples of *P. fluviatilis*. Light blue indicates little to no genetic differentiation between the two samples. “×” indicates that *p* is more than 0.05.

Pairwise F_ST_ estimates among 15 *R. rutilus* and 13 *P. fluviatilis* populations are presented in Figure 2 and Tables S2 and S3. Negative F_ST_ values were set to zero, as they are generally considered to reflect sampling variance rather than biologically meaningful differentiation. In *R. rutilus*, F_ST_ values ranged from 0 to 0.269. Most comparisons showed low to moderate differentiation (median F_ST_ = 0.072), with 41% of values below 0.05 and only a single comparison (1%) exceeding 0.25. Statistically significant differentiation (*p* < 0.05) was detected in 41% of comparisons.

In *P. fluviatilis*, F_ST_ values ranged from 0 to 0.816, indicating a wider range of genetic differentiation among populations. A total of 41% of pairwise comparisons were statistically significant. Approximately 40% of comparisons exceeded F_ST_ > 0.15, and the highest values were observed in comparisons involving the PLA sampling site. Overall, *P. fluviatilis* exhibited a broader distribution of F_ST_ values compared to *R. rutilus* (Figure 2). No corresponding population pairs exhibited high differentiation (F_ST_ > 0.25) in both species. Five comparisons (ANT/NEM, DRU/KUR, DRU/NEM, KAU/NEM, and NEM/SMA) were statistically significant in both species.

Hierarchical AMOVA with populations grouped a priori by drainage basin showed that the among-basin component of variation was low and non-significant in both species (Table 2). In *R. rutilus*, basins accounted for only 2.62% of the total molecular variance (F_CT_ = 0.026, *p* = 0.1880), with most variation residing within populations (89.01%; F_ST_ = 0.110, *p* < 0.0001) and a small but significant fraction among populations within basins (8.37%; F_SC_ = 0.086, *p* < 0.0001). In *P. fluviatilis*, overall and within-group differentiation was considerably stronger (F_ST_ = 0.285, *p* < 0.0001; F_SC_ = 0.207, *p* < 0.0001), yet the among-basin component remained non-significant (F_CT_ = 0.099, *p* = 0.086), explaining only 9.91% of the variance. Thus, although *P. fluviatilis* was markedly more structured than *R. rutilus*, in neither species did drainage-basin boundaries account for a significant share of genetic variation.

### 3.3. Haplotype Networks and Phylogenetic Relationships

**Figure 3:**
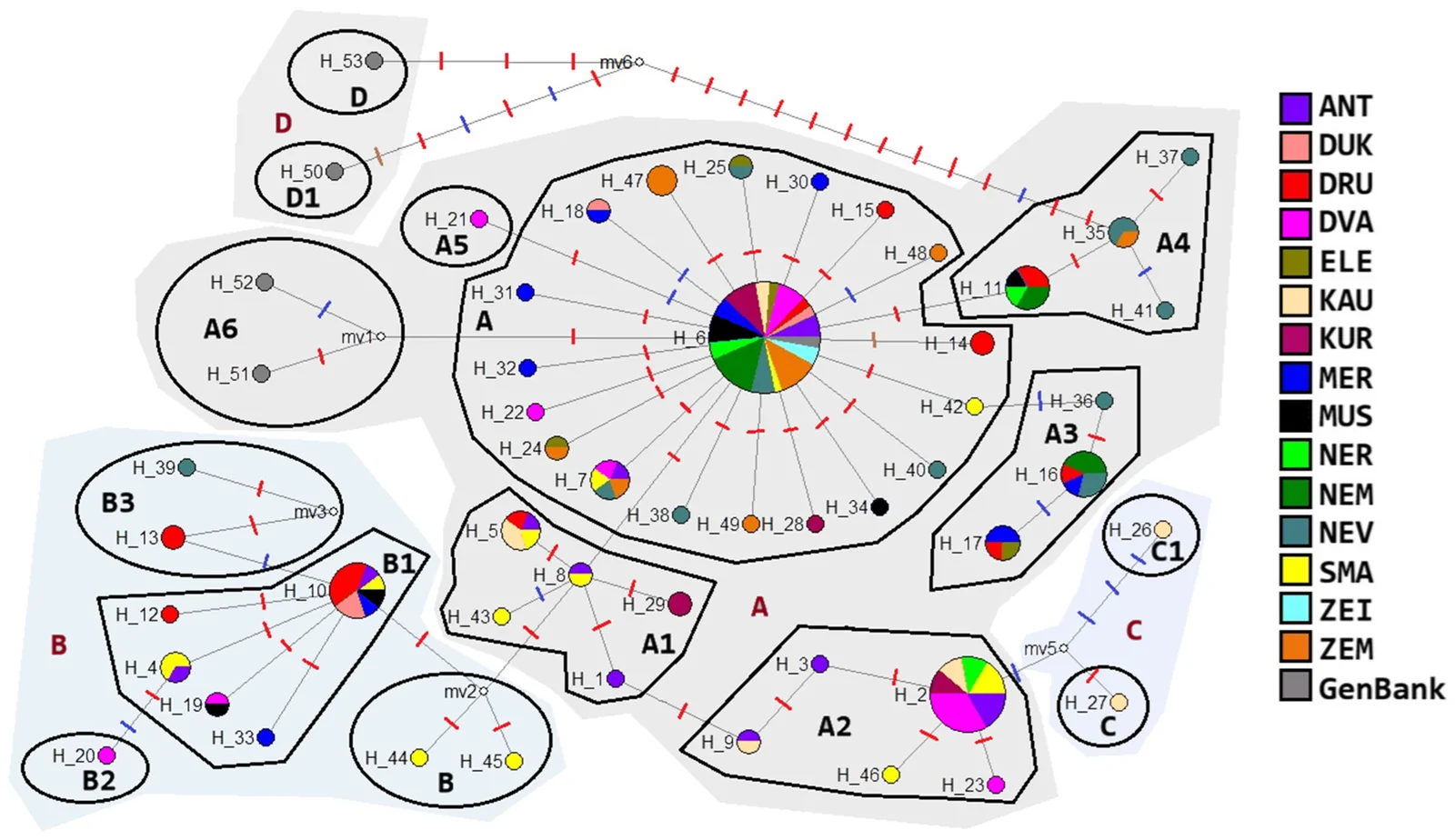
Median-joining haplotype of *R. rutilus*. Circles represent haplotypes, with sizes proportional to their frequencies. Pie charts indicate the distribution of haplotypes among sampling locations (see legend). Grey colour denotes haplotypes originating from GenBank. Major haplotype clusters are indicated by dark red capital letters (A–D), while black alphanumeric labels (e.g., A, A1, B2) denote subclusters. These clusters are defined based on network topology for descriptive purposes only and in most cases do not represent phylogenetically supported lineages. Hatch marks on branches represent mutational steps, with red, blue, and brown ticks corresponding to transitions, transversions, and indels, respectively. “mv” denotes median vectors.

**Figure 4:**
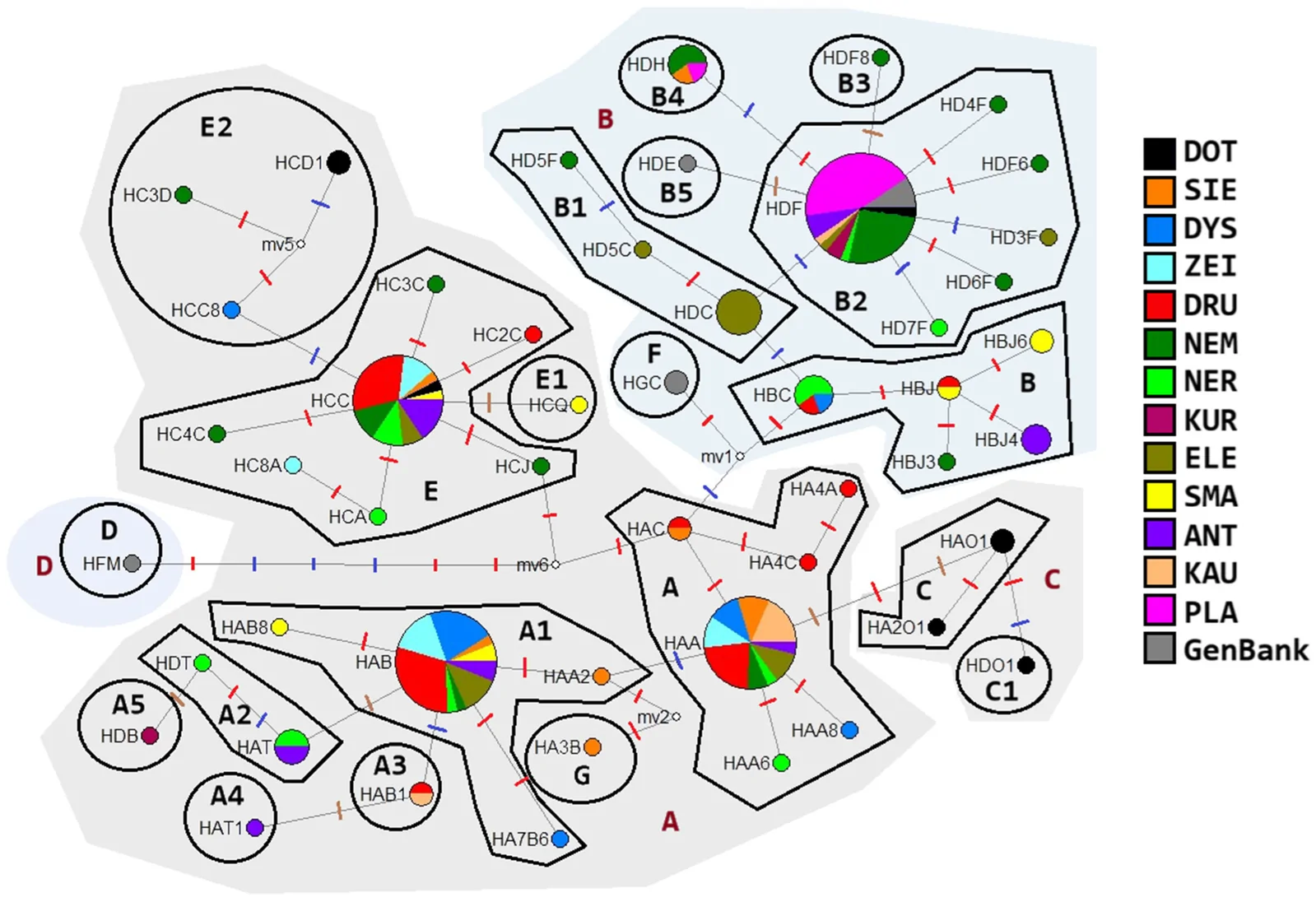
Median-joining haplotype network of *P. fluviatilis*. Circles represent haplotypes, with sizes proportional to their frequencies. Pie charts indicate the distribution of haplotypes among sampling locations (see legend). Grey colour denotes haplotypes originating from GenBank. Major haplotype clusters are indicated by dark red capital letters (A–D), while black alphanumeric labels (e.g., A, A1, B2) denote subclusters. These clusters are defined based on network topology for descriptive purposes only and in most cases do not represent phylogenetically supported lineages. Hatch marks on branches represent mutational steps, with red, blue, and brown ticks corresponding to transitions, transversions, and indels, respectively. “mv” denotes median vectors.

The median-joining haplotype networks based on concatenated mtDNA ATP6–D-loop sequences revealed contrasting patterns of haplotype distribution between the two species (Figure 3 and Figure 4; Table S4). In *R. rutilus*, the network exhibited a pronounced star-like structure centred on a single dominant haplotype (H_6), which comprised 98 sequences and was detected across all Lithuanian sampling sites as well as in additional GenBank records. Most other haplotypes differed from H_6 by a single mutational step, indicating low sequence divergence. Most haplotypes were rare and often represented by single occurrences. Haplotypes were distributed across sampling locations without clear geographic structuring. All distinct ATP6 and D-loop sequences were deposited in GenBank (PX614649–PX614669; PX614670–PX614695, respectively).

In *P. fluviatilis*, the haplotype network showed a less centralized structure, with multiple common haplotypes (e.g., HDF, HAB, HAA, HCC) and no single haplotype dominating across all sampling sites. These haplotypes were distributed across different locations, and several network-defined clusters contained multiple moderately frequent haplotypes. Compared to *R. rutilus*, haplotypes were less frequently shared among sampling locations, and the network exhibited a more heterogeneous configuration. All distinct ATP6 and D-loop sequences forming composite haplotypes were submitted to GenBank (PX766250–PX766268; PX705063–PX705093, respectively).

In both species, haplotypes were visually grouped into clusters (A–D) based on network topology. However, these groupings are descriptive and do not represent phylogenetically supported lineages. Most samples were assigned to two major clusters (A and B), whereas the remaining clusters contained only a small number of haplotypes, often represented exclusively by sequences obtained from GenBank. Mutational differences among haplotypes were predominantly transitions, with fewer transversions and a small number of insertion–deletion (InDel) events. The *R. rutilus* network included some longer branches connecting peripheral haplotypes, whereas the *P. fluviatilis* network was characterized by shorter mutational distances but a more complex branching structure.

The Maximum Likelihood phylogenetic trees (Figures S1 and S2) revealed shallow topologies with short branch lengths and low genetic divergence among haplotypes in both species. Although several nodes received moderate bootstrap support (≥70%), these did not correspond to well-defined or deeply divergent clades, and no clear phylogenetically supported lineages were identified.

### 3.4. Demographic Analyses

Neutrality test results for concatenated mtDNA ATP6–D-loop sequences are presented in Table 3. For the pooled dataset of *R. rutilus*, Tajima’s D was negative but not statistically significant (D = −0.835, *p* = 0.273), whereas Fu’s Fs was significantly negative (Fs = −3.291, *p* = 0.002). In *P. fluviatilis*, both Tajima’s D (D = −0.039, *p* = 0.607) and Fu’s Fs (Fs = −0.798, *p* = 0.215) were negative but not statistically significant.

**Figure 5:**
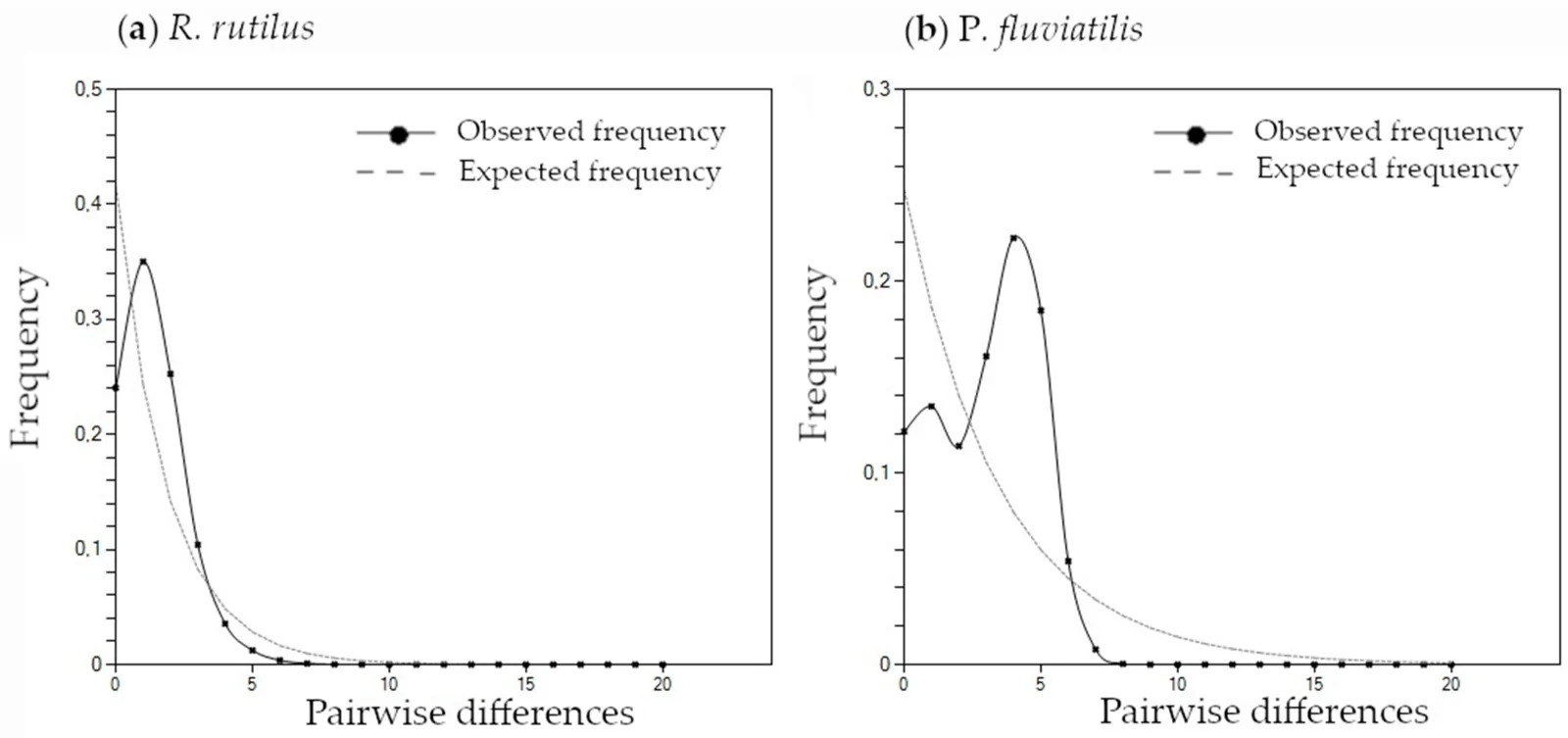
Mismatch distribution analysis showing observed (solid line with dots) and expected (dashed line) distributions of pairwise differences for concatenated mtDNA ATP6–D-loop sequences of: (**a**) *R. rutilus* and (**b**) *P. fluviatilis*.

Mismatch distribution analysis (Figure 5) revealed contrasting patterns between the two species. In *R. rutilus*, the observed distribution was broadly unimodal. However, the least-squares procedure used to fit the sudden demographic expansion model did not converge, preventing reliable estimation of goodness-of-fit statistics (SSD and Harpending’s raggedness index). In *P. fluviatilis*, the observed mismatch distribution was bimodal. The fit of the observed distribution to demographic and spatial expansion models was assessed using SSD and Harpending’s raggedness index. Neither the demographic expansion model (SSD = 0.0106, *p* = 0.197; raggedness = 0.0247, *p* = 0.381) nor the spatial expansion model (SSD = 0.0089, *p* = 0.539; raggedness = 0.0247, *p* = 0.777) was rejected. Overall, the contrasting mismatch profiles are consistent with the neutrality tests: the unimodal distribution and significantly negative Fu’s Fs in *R. rutilus* support recent population expansion, whereas the bimodal distribution in *P. fluviatilis* is more consistent with the presence of divergent mitochondrial lineages or population structure than with a single sudden expansion.

**Figure 6:**
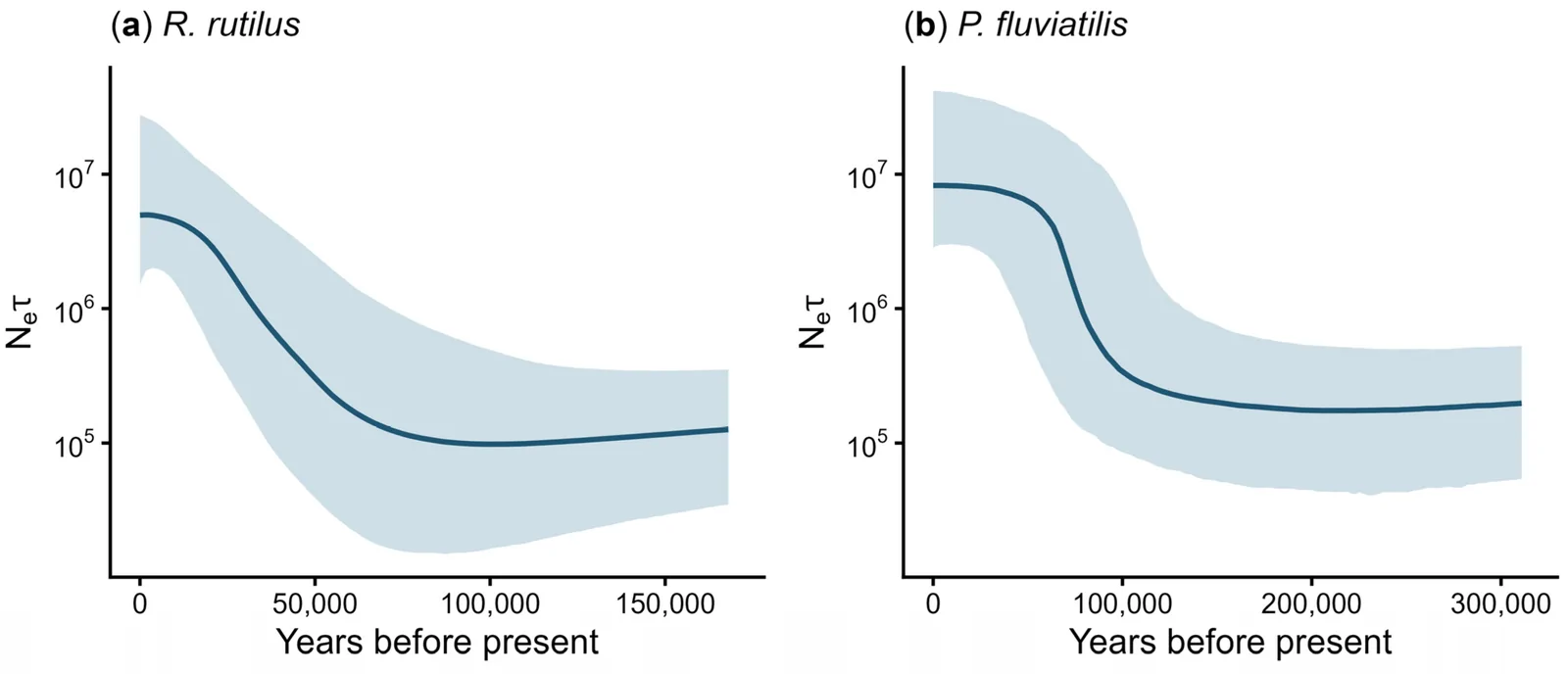
Bayesian skyline plots of effective population size (Neτ) through time for (**a**) *R. rutilus* and (**b**) *P. fluviatilis*. Solid lines show median estimates; shaded areas show the 95% HPD intervals.

Bayesian skyline plot (BSP) analyses (Figure 6) recovered a signal of historical population growth in both species, with effective population size (Neτ) rising from low ancestral values to a higher plateau. In both *R. rutilus* and *P. fluviatilis* the increase occurred largely during the Late Pleistocene, approximately 50,000–100,000 years before present, after which Neτ remained relatively stable toward the present. The expansion was more pronounced in *P. fluviatilis*, whereas *R. rutilus* showed a more gradual increase over a shallower time depth. In both species, the 95% HPD intervals were wide, particularly for older time periods, indicating substantial uncertainty in the magnitude and exact timing of these demographic changes.

Overall, the two species exhibited contrasting patterns of genetic structure. *R. rutilus* showed widespread haplotype sharing, low genetic differentiation, and weak spatial structure, whereas *P. fluviatilis* displayed higher differentiation and more pronounced population structuring. Consistent with this, the neutrality tests and mismatch distributions provided stronger evidence of population expansion in *R. rutilus* than in *P. fluviatilis*.

## 4. Discussion

The present study revealed contrasting patterns of genetic structure between *R. rutilus* and *P. fluviatilis* despite their co-occurrence in the same Lithuanian freshwater systems and the use of the same mtDNA ATP6–D-loop region. Contrary to our first hypothesis, genetic differentiation was not consistently associated with major drainage-basin boundaries in either species. Consistent with our second hypothesis, *P. fluviatilis* exhibited stronger and more spatially heterogeneous genetic structuring than *R. rutilus*. Together, these results indicate that contemporary hydrological boundaries alone do not explain the observed genetic variation. Species-specific ecological differences may help explain the contrasting genetic structure detected in the two species, although other historical and demographic processes may also contribute to these patterns.

### 4.1. Comparative Population Genetic Structure

While both species showed relatively high haplotype diversity and star-like haplotype networks, the extent and configuration of these networks differed between them. In *R. rutilus*, the network had a pronounced star-like topology centred on a widespread haplotype, with numerous low-frequency haplotypes connected to it by single mutational steps. This configuration is often associated with demographic expansion [46] or high gene flow [47], although these explanations cannot be distinguished with the present data alone. *P. fluviatilis*, by contrast, showed a less pronounced star-like topology, with several moderately frequent haplotypes forming secondary centres within a more subdivided network. In neither species did Maximum Likelihood analyses recover well-supported monophyletic lineages. The trees were shallow, with short branches and no strongly supported internal clades, mirroring the few mutational steps seen in the networks. The visually defined network clusters therefore should not be interpreted as independently evolving lineages, highlighting the value of combining phylogenetic and network approaches when interpreting such patterns [48,49]. In both species, the shallow phylogenetic signal supports interpretation at the level of population structure rather than deeper evolutionary history. Population-level analyses reinforced this contrast. In *R. rutilus*, low and largely non-significant pairwise differentiation, combined with the wide distribution of common haplotypes across basins, is consistent with ongoing gene flow, recent shared ancestry, or both [47,48]. *P. fluviatilis* was considerably more structured overall, yet—as in *R. rutilus*—this differentiation was not organised by drainage basins, with the among-basin component non-significant in both species.

*R. rutilus* occupies a broad range of freshwater habitats and uses different areas for feeding, overwintering, and spawning, while also undertaking seasonal movements [50,51,52]. Where water bodies are hydrologically connected, these movement patterns may facilitate gene flow among neighbouring populations [10]. Mark–recapture studies have also documented strong homing to spawning areas, showing that mobility and site fidelity can coexist in *R. rutilus* [52]. *P. fluviatilis* likewise performs seasonal movements between overwintering and spawning habitats, but its spawning migrations are generally shorter than those of *R. rutilus*. It shows strong site fidelity and homing and often completes its life cycle within a single water body [15]. Within individual lakes, *P. fluviatilis* can exhibit fine-scale ecological and behavioural differentiation, reflecting individual specialization in habitat use [53]. Genetic differentiation among *P. fluviatilis* populations within a single lake has also been documented [7]. The contrast should therefore not be viewed simply as a difference between a mobile and a sedentary species, but rather as differences in habitat use, movement among connected habitats, and spawning-site fidelity. These differences may affect gene flow and help explain the finer-scale genetic structure observed in *P. fluviatilis*.

The stronger structuring observed in *P. fluviatilis* is consistent with earlier reports of substantial mitochondrial differentiation among European perch populations from countries geographically close to Lithuania. Using a concatenated mitochondrial dataset comprising four markers (Cytb, D-loop, 16S, and COI), Vanina et al. [54] found significant differentiation among three allopatric populations from Poland, the Czech Republic, and Slovakia (overall *F*_ST_ = 0.228, *p* < 0.01; 22.8% of the molecular variance among populations and 77.2% within), whereas a broader comparison of seven Central- and North-European populations detected even stronger structure (*F*_ST_ = 0.501, *p* < 0.01), largely driven by the pronounced divergence of the Finnish populations [55]. These estimates are higher than those obtained in our study, but the studies compared populations separated by much greater geographic distances, and the seven-population study additionally included strongly divergent Finnish populations, whereas our sampling was restricted to Lithuanian freshwater systems. The comparison should therefore not be interpreted as evidence that geographic separation alone determines genetic structure; rather, both datasets indicate that *P. fluviatilis* can exhibit marked mitochondrial differentiation among populations, and our results extend this finding by showing that such structure need not follow drainage-basin boundaries.

These ecological differences may help explain the contrasting genetic structure observed in the two species, but they are unlikely to be the only factors involved. Human-mediated processes such as stocking may also alter natural patterns of genetic differentiation and obscure historical population structure. In *R. rutilus*, a recent stocking event was shown to disrupt the expected isolation-by-distance pattern within a river population [10]. Because stocking histories were not available for the sampled Lithuanian populations, this possibility cannot be evaluated directly and should be regarded as an additional rather than a primary explanation.

### 4.2. Demographic Signals

The two species also differed in their demographic signals, again more in strength and concordance than in kind. In *R. rutilus*, a negative Tajima’s *D* [40], a significantly negative Fu’s *Fs* [41], and a broadly unimodal mismatch distribution [42] were individually consistent with demographic expansion. The Bayesian skyline plot (BSP) further indicated an increase in effective population size during the Late Pleistocene (~50,000–100,000 years before present), with little subsequent change toward the present, although the wide HPD intervals indicate substantial uncertainty. These signals should not be overinterpreted. The mismatch model failed to converge, so the observed unimodal distribution cannot be taken as a formal fit to a sudden-expansion model. Moreover, comparable signatures are widespread across a range of taxa and are not uniquely diagnostic of expansion [56,57,58], as they may also arise from bottlenecks, population structure, or selection acting on mtDNA [59]. In *P. fluviatilis*, the evidence was weaker and less concordant: a non-significant Tajima’s D and Fu’s Fs, a bimodal mismatch distribution [42], and a BSP increase toward the present, although the BSP estimate carried considerable uncertainty. Taken together, these results do not support a single demographic scenario for either species, and the BSP estimates are best interpreted as broad historical signals rather than precise reconstructions of demographic change. The contrasting demographic signals may also reflect differences in postglacial history and historical population subdivision. Range-wide analyses of *R. rutilus* indicate large, stable populations with high long-term effective population sizes and evidence of demographic expansion during the Middle Pleistocene, whereas the Late Pleistocene glacial maxima appear to have left a comparatively limited demographic imprint [60]. Such historical expansion, together with the retention and widespread distribution of shared haplotypes, may have contributed to the weaker contemporary genetic structure observed in roach in this study. *P. fluviatilis*, by contrast, may have followed a more subdivided refugial and recolonisation history, as previous phylogeographic work has linked geographic differentiation in this species to glacial refugia and postglacial colonisation [25]. Some of the stronger differentiation observed here may therefore reflect historical processes that predate the current Lithuanian drainage configuration.

The two species may also differ in long-term effective population size, which could affect the rate at which genetic drift generates differentiation and the persistence of geographically restricted haplotypes. Life-history differences may contribute to such variation: both species are highly fecund, externally fertilising spring spawners whose egg output increases with female body size (reaching almost 78,000 eggs in large *P. fluviatilis* females [61] and over 30,000 in *R. rutilus* [62]). Differences in reproductive output and age at maturity may therefore influence demographic turnover and effective population size rather than gene flow directly. Our BSP analyses, however, characterise changes in effective population size through time rather than long-term differences between the species, which therefore remain a possible but untested contributor to the contrasting structure of the two species [63].

Interpreted alongside the structural results, these demographic signals are broadly consistent with the population-genetic patterns. In *R. rutilus*, weak differentiation and widespread haplotype sharing may reflect not only contemporary gene flow but also demographic and range expansion, which can homogenise genetic variation across a region [64]. The more heterogeneous structure of *P. fluviatilis*, by contrast, aligns with its less concordant demographic history and more localised population dynamics. Together, these results are compatible with a contribution of Late Pleistocene processes to the observed patterns, although the strength and coherence of the signals differ between the two species.

### 4.3. Conservation Implications and Future Directions

The contrasting patterns indicate that co-occurring freshwater species can respond differently to the same landscape, with direct relevance for conservation and fisheries management. The stronger structuring observed in *P. fluviatilis* than in *R. rutilus* suggests that species with more limited dispersal may be more sensitive to habitat fragmentation and loss of connectivity. Management units defined from hydrology alone may therefore fail to capture biologically meaningful connectivity for every species [11], and conservation planning should account for species-specific differences in dispersal and population structure rather than assuming concordant responses among co-occurring taxa [65,66]. Because the present data cannot resolve the relative contributions of ecology, historical demography, effective population size, and anthropogenic translocation, however, these implications are best treated as precautionary. Species-specific genetic information is most useful when integrated with movement ecology, habitat connectivity, and stocking history rather than applied based on drainage boundaries or genetic differentiation alone.

The interpretation of these findings is also limited by the nature of the genetic markers used. Because mitochondrial markers reflect only maternally inherited lineages, sex-biased dispersal could make the observed mtDNA structure appear stronger than the underlying population structure. More fundamentally, although we combined two mitochondrial regions (ATP6 and the D-loop), both are part of the same maternally inherited, non-recombining mitochondrial genome and therefore capture only part of each species’ evolutionary history [67,68]. Sampling also spanned an extended period (2009–2025). Although temporal variation in population genetic structure cannot be completely excluded, substantial changes in mitochondrial haplotype frequencies are unlikely over this relatively short ecological timescale.

Incorporating nuclear or genome-wide markers, together with broader geographic and temporal sampling across Lithuanian and neighbouring Baltic populations, would allow these processes to be distinguished and would substantially improve resolution of population connectivity, demographic history, and evolutionary dynamics across Baltic freshwater systems [69]. Integrating nuclear genetic data with movement ecology, historical stocking records, and measures of environmental connectivity would help determine whether the stronger differentiation of *P. fluviatilis* reflects species-specific dispersal, historical population subdivision, differences in effective population size, or human-mediated changes in connectivity, and would provide a firmer basis for defining biologically meaningful management units in Baltic freshwater fishes.

## 5. Conclusions

By applying an identical marker set and analytical framework to two co-occurring but ecologically contrasting fishes within the same hydrological landscape, this study was able to separate the influence of shared drainage configuration from that of species-specific ecology. In neither *R. rutilus* nor *P. fluviatilis* did genetic differentiation align consistently with major drainage-basin boundaries, indicating that contemporary hydrological divisions alone do not determine genetic variation. Instead, the two species differed in both the magnitude and spatial pattern of differentiation. *P. fluviatilis* was more structured and spatially heterogeneous, whereas *R. rutilus* was weakly structured, with widespread haplotype sharing consistent with gene flow or recent expansion. This discordance suggests that dispersal ability and ecological differences, rather than drainage configuration, are the main drivers of population structure in these systems.

This interspecific contrast is best regarded as a general tendency rather than an absolute distinction, and the inferences are bounded by reliance on a single, maternally inherited mitochondrial locus and geographically restricted sampling. Integrating nuclear or genome-wide data, broader geographic coverage, and model-based demographic analyses will be needed to confirm these patterns and to resolve the processes shaping genetic diversity in Baltic freshwater fishes.

## Figures and Tables

**Table 1:** Quantitative genetic diversity parameters of *R. rutilus* and *P. fluviatilis* samples in Lithuania.

Samples	*n*	*S*	*h*	*Hd* ± SD	*π* ± SD	*k*
*R. rutilus*						
ANT	18	5	10	0.843 ± 0.077	0.0017 ± 0.0003	1.379
DUK	6	2	3	0.733 ± 0.155	0.0011 ± 0.0003	0.867
DRU	18	8	10	0.922 ± 0.051	0.0020 ± 0.0003	1.634
DVA	20	10	8	0.774 ± 0.071	0.0018 ± 0.0004	1.495
ELE	6	4	4	0.800 ± 0.172	0.0016 ± 0.0005	1.333
KAU	11	7	6	0.855 ± 0.085	0.0024 ± 0.0007	1.927
KUR	15	4	4	0.552 ± 0.137	0.0011 ± 0.0003	0.876
MER	15	7	9	0.848 ± 0.088	0.0018 ± 0.0003	1.467
MUS	12	4	5	0.576 ± 0.163	0.0010 ± 0.0003	0.803
NER	8	2	3	0.607 ± 0.164	0.0008 ± 0.0003	0.679
NEM	20	2	3	0.426 ± 0.122	0.0006 ± 0.0002	0.458
NEV	19	9	11	0.865 ± 0.071	0.0024 ± 0.0004	1.936
SMA	16	8	12	0.958 ± 0.036	0.0025 ± 0.0003	2.075
ZEI	5	0	1	0	0	0
ZEM	20	7	7	0.637 ± 0.116	0.0011 ± 0.0003	0.868
Total	209	37	49	0.760 ± 0.031	0.0017 ± 0.0001	1.402
*P. fluviatilis*						
DOT	8	12	6	0.929 ± 0.084	0.0046 ± 0.0007	5.464
SIE	9	8	7	0.917 ± 0.091	0.0023 ± 0.0006	2.722
DYS	14	9	6	0.736 ± 0.109	0.0023 ± 0.0005	2.143
DRU	31	10	10	0.811 ± 0.043	0.0026 ± 0.0002	2.443
ZEI	12	5	4	0.758 ± 0.081	0.0022 ± 0.0003	2.121
ELE	19	9	7	0.819 ± 0.063	0.0030 ± 0.0003	2.854
KAU	7	7	3	0.524 ± 0.209	0.0021 ± 0.0010	2.000
ANT	16	10	7	0.883 ± 0.047	0.0039 ± 0.0003	4.067
NER	15	10	10	0.933 ± 0.047	0.0036 ± 0.0003	3.762
SMA	8	10	6	0.929 ± 0.086	0.0046 ± 0.0006	4.571
KUR	3	4	2	0.667 ± 0.314	0.0028 ± 0.0013	2.667
NEM	30	16	15	0.857 ± 0.063	0.0034 ± 0.0004	3.299
PLA	19	2	2	0.105 ± 0.092	0.0002 ± 0.0002	0.211
Total	191	33	46	0.892 ± 0.012	0.0032 ± 0.0001	3.619

Notes: *n*: sample size; *S*: number of polymorphic sites, *h*: number of haplotypes, *Hd*: haplotype diversity, *π*: nucleotide diversity, *k*: average number of nucleotide differences.

**Table 2:** Hierarchical analysis of molecular variance (AMOVA) for concatenated mtDNA ATP6–D-loop sequences of *R. rutilus* (209 individuals, 15 populations, three drainage basins) and *P. fluviatilis* (191 individuals, 13 populations, two drainage basins), with sampling sites assigned to drainage basins. Va, Vb, and Vc are the variance components among basins, among populations within basins, and within populations, respectively. Fixation indices (F_CT_, among basins; F_SC_, among populations within basins; F_ST_, overall) and their significance levels are shown.

Source of Variation	df	Sum of Squares	Variance Components	Percentage Variation	Fixation Indices	*p*-Value
*R. rutilus*						
Among basins (Nemunas, Daugava, Lielupe ^1^)	2	4.499	0.019 Va	2.620	F_CT_ = 0.026 F_SC_ = 0.086 F_ST_ = 0.110	0.188 <0.001 <0.001
Among populations within basins	12	17.554	0.060 Vb	8.370		
Within populations	194	123.723	0.638 Vc	89.010		
Total	208	145.775	0.717	100.000		
*P. fluviatilis*						
Among basins (Nemunas, Daugava)	1	23.010	0.185 Va	9.910	F_CT_ = 0.099 F_SC_ = 0.207 F_ST_ = 0.285	0.086 <0.001 <0.001
Among populations within basins	11	66.941	0.348 Vb	18.630		
Within populations	178	237.599	1.335 Vc	71.470		
Total	190	327.550	1.868	100.000		

^1^. Lielupe was represented by a single *R. rutilus* site. *P. fluviatilis* was not sampled in the Lielupe or Venta basins. Basin assignments follow Table S1.

**Table 3:** Neutrality test statistics (Tajima’s D and Fu’s Fs) for concatenated mtDNA ATP6–D-loop sequences of *R. rutilus* and *P. fluviatilis*.

Species	Tajima’s D	*p*-Value	Fu’s Fs	*p*-Value
*R. rutilus*	−0.835	0.273	−3.291	0.002
*P. fluviatilis*	−0.039	0.607	−0.798	0.215

## Data Availability

The datasets involved in this study have been deposited in GenBank under the accession numbers PX614649–PX614669, PX614670–PX614695, PX766250–PX766268, and PX705063–PX705093.

## Supplementary Materials

The following supporting information can be downloaded at: https://www.mdpi.com/article/10.3390/ani16162540/s1, Table S1. Information on the collection locations, years, and associated basins for both species. Table S2. Genetic differentiation of *R. rutilus* samples. Pairwise F_ST_ comparisons and the statistical significance of these values (*p*) are presented below and above the diagonal, respectively. Table S3. Genetic differentiation of *P. fluviatilis* samples. Pairwise F_ST_ comparisons and the statistical significance of these values (*p*) are presented below and above the diagonal, respectively. Table S4. Distribution of *R. rutilus* and *P. fluviatilis* haplotypes and network-defined clusters across sampling locations. Figure S1. Maximum Likelihood phylogenetic tree of *R. rutilus* haplotypes inferred using the HKY+G nucleotide substitution model. The tree was rooted using *Scardinius erythrophthalmus* as an outgroup. Only bootstrap values ≥ 70% are shown. Figure S2. Maximum Likelihood phylogenetic tree of *P. fluviatilis* haplotypes inferred using the HKY+I nucleotide substitution model. The tree was rooted using *Perca flavescens* as an outgroup. Only bootstrap values ≥ 70% are shown.

## Abbreviations

The following abbreviations are used in this manuscript:

mtDNA	mitochondrial DNA
PCR	Polymerase Chain Reaction
*S*	polymorphic sites
*h*	number of haplotypes
*Hd*	haplotype diversity
*π*	nucleotide diversity
*k*	average number of nucleotide differences
mv	median vectors
InDel	insertion–deletion
ML	Maximum Likelihood
SSD	sum of squared deviations
BSP	Bayesian skyline plot
MCMC	Markov Chain Monte Carlo
ESS	effective sample sizes
Neτ	effective population size
