# Supplementary material for: Comparative Population Genetics of Rutilus rutilus and Perca fluviatilis in Lithuanian Freshwater Ecosystems
Source: Animals (Basel). 2026 Aug 14;16(16):2540. doi: 10.3390/ani16162540 (PMC13508947; doi:10.3390/ani16162540)
Supplement: Supplementary file 1 [file animals-16-02540-s001.zip › animals-4472850-supplementary.pdf]

Table S1. Information on the collection locations, years, and associated basins for both species.

**Sampling Sites (Codes)**<br>*Geographic Coordinates*	***Basin* / Sub-basin**	**Species**	**Sample Size**	**Sample Year**
**Antalieptė Reservoir (ANT)**<br>*55°37′12.5″N 26°00′35.9″E*	***Nemunas River*** / Šventoji River	*R. rutilus*<br>*P. fluviatilis*	18<br>18	2025
**Lake Drūkšiai (DRU)**<br>*55°38′45.7″N 26°35′53.3″E*	***Daugava River*** / Dysna River	*R. rutilus*<br>*P. fluviatilis*	10, 8<br>22, 9	2022, 2025<br>2009, 2022
**Elektrėnai Reservoir (ELE)**<br>*54° 45′ 59.28″, 24° 40′ 59.75″*	***Nemunas River*** / Strėva River	*R. rutilus*<br>*P. fluviatilis*	6<br>19	2018<br>2017
**Kaunas Reservoir (KAU)**<br>*54°50′51.5″N 24°12′27.2″E*	***Nemunas River*** / Nemunas River	*R. rutilus*<br>*P. fluviatilis*	11<br>7	2025
**Curonian Lagoon (KUR)**<br>*55°21′46.4″N 21°15′46.5″E*	***Baltic Sea*** / Nemunas River	*R. rutilus*<br>*P. fluviatilis*	15<br>3	2025<br>2017
**Neris River (NER)**<br>*54°57′24.8″N 23°55′41.9″E*<br>*54°51′45.0″N 25°24′40.8″E*	***Nemunas River*** / Neris River	*R. rutilus*<br>*P. fluviatilis*	8<br>10, 5	2022<br>2009, 2010
**Nemunas River (NEM)**<br>*54°53′34.6″N 23°53′53.5″E*<br>*55° 17′ 50.85″, 21° 22′ 53.4″*	***Nemunas River*** / Nemunas River	*R. rutilus*<br>*P. fluviatilis*	20<br>14, 18	2022<br>2009, 2010
**Lake Smalvas (SMA)**<br>*55°37′20.6″N 26°23′21.9″E*	***Daugava River*** / Dysna River	*R. rutilus*<br>*P. fluviatilis*	16<br>8	2025
**Lake Žeimenys (ZEI)**<br>*55° 15′ 59.78″, 26° 1′ 50.13″*	***Nemunas River*** / Žeimena River	*R. rutilus*<br>*P. fluviatilis*	5<br>12	2017<br>2018
**Lake Dūkštas (DUK)**<br>*55°34′57.2″N 26°19′29.1″E*	***Daugava River*** / Dysna River	*R. rutilus*	6	2019
**Lake Dvarčionys (DVA)**<br>*54°43′28.9″N 25°22′21.0″E*	***Nemunas River*** / Neris River	*R. rutilus*	20	2018
**Merkys River (MER)**<br>*54°06′28.4″N 24°16′35.3″E*	***Nemunas River*** / Merkys River	*R. rutilus*	15	2022
**Mūša River (MUS)**<br>*56°15′09.3″N 24°22′16.1″E*	***Lielupė River*** / Mūša River	*R. rutilus*	12	2022

**Nevėžis River (NEV)** *54°57′53.5″N 23°47′58.2″E*	***Nemunas River*** / Nevėžis River	*R. rutilus*	19	2022
**Žeimena River (ZEM)** *55°14′26.6″N 25°58′48.3″E*	***Nemunas River*** / Žeimena River	*R. rutilus*	20	2022
**Dotnuvelė River (DOT)** *55° 18′ 35.63″, 23° 58′ 28.14″*	***Nemunas River*** / Nevėžis River	*P. fluviatilis*	8	2014
**Siesartis River (SIE)** *55° 17′ 28.39″, 24° 51′ 42.06″*	***Šventoji River*** / Siesartis River	*P. fluviatilis*	9	2015
**Lake Dysnai (DYS)** *55°29′21.4″N 26°22′11.7″E*	***Daugava River*** / Dysna River	*P. fluviatilis*	14	2013
**Lake Plateliai (PLA)** *56°02′40.1″N 21°52′22.2″E*	***Nemunas River*** / Minija River	*P. fluviatilis*	19	2010

Table S2. Genetic differentiation of *R. rutilus* samples. Pairwise F_{ST} comparisons and the statistical significance of these values (P) are presented below and above the diagonal, respectively.

	***R. rutilus* samples**	**ANT**	**DUK**	**DRU**	**DVA**	**ELE**	**KAU**	**KUR**	**MER**	**MUS**	**NER**	**NEM**	**NEV**	**SMA**	**ZEI**	**ZEM**
1	ANT		0.12623	0.00515	0.37184	0.06306	0.55410	0.17404	0.00069	0.01881	0.50797	0.00000	0.00020	0.24116	0.29631	0.00010
2	DUK	0.07787		0.84823	0.11761	0.69924	0.03742	0.09930	0.72458	0.68360	0.10949	0.02208	0.08762	0.06960	0.45015	0.05237
3	DRU	0.10601	0.00000		0.00158	0.21156	0.00188	0.00267	0.19364	0.18226	0.06247	0.00891	0.01614	0.00356	0.23612	0.00040
4	DVA	0.00114	0.07743	0.11217		0.08573	0.25275	0.05970	0.00119	0.04019	0.88338	0.00040	0.00000	0.06564	0.32215	0.00069
5	ELE	0.09348	0.01000	0.04452	0.08905		0.04049	0.14979	0.82675	0.19107	0.23819	0.22483	0.52351	0.01287	0.99990	0.37125
6	KAU	0.00000	0.13328	0.16159	0.00984	0.10095		0.07742	0.00020	0.00198	0.37016	0.00030	0.00050	0.21978	0.26324	0.00000
7	KUR	0.03042	0.09783	0.12087	0.05319	0.05017	0.06431		0.01247	0.08534	0.45174	0.01020	0.00257	0.00931	0.99990	0.02515
8	MER	0.11333	0.00000	0.02129	0.11485	0.00000	0.14912	0.07519		0.22988	0.09266	0.10999	0.02267	0.00010	0.74260	0.00168
9	MUS	0.08972	0.00000	0.02828	0.07860	0.03835	0.15087	0.04857	0.02621		0.32126	0.14296	0.04445	0.00168	0.89100	0.15949
10	NER	0.00000	0.12347	0.08667	0.00000	0.05040	0.00288	0.00000	0.06106	0.03491		0.11296	0.10039	0.09326	0.48926	0.14365
11	NEM	0.15794	0.17168	0.10473	0.14358	0.03649	0.21890	0.07728	0.03945	0.04178	0.07237		0.04287	0.00000	0.72339	0.14335
12	NEV	0.15926	0.08491	0.07754	0.14758	0.00000	0.17433	0.12534	0.07189	0.07710	0.06642	0.06479		0.00000	0.29413	0.01109
13	SMA	0.01864	0.09783	0.11717	0.04766	0.15982	0.02317	0.12273	0.17659	0.16206	0.07911	0.26919	0.21414		0.04287	0.00000
14	ZEI	0.03413	0.09513	0.04296	0.02806	0.00000	0.07121	0.00000	0.00000	0.00000	0.01809	0.00000	0.02520	0.13233		0.86477
15	ZEM	0.12291	0.08665	0.13033	0.11822	0.01097	0.17207	0.04849	0.07240	0.02481	0.03724	0.03890	0.09565	0.22953	0.00000	

Pairwise F_{ST} comparisons and the statistical significance of these values (P) are presented below and above the diagonal, respectively.

Table S3. Genetic differentiation of *P. fluviatilis* samples. Pairwise F_{ST} comparisons and the statistical significance of these values (P) are presented below and above the diagonal, respectively.

***P. fluviatilis* samples**		**DOT**	**SIE**	**DYS**	**DRU**	**ZEI**	**ELE**	**KAU**	**ANT**	**NER**	**SMA**	**KUR**	**NEM**	**PLA**
1	DOT		0.27562	0.01297	0.06257	0.10553	0.10058	0.19909	0.23305	0.34313	0.23117	0.11642	0.01040	0.00000
2	SIE	0.03365		0.37155	0.70161	0.66330	0.08781	0.65429	0.28690	0.42293	0.24770	0.01802	0.00099	0.00000
3	DYS	0.17883	0.00151		0.08811	0.31977	0.00277	0.06089	0.02960	0.01742	0.05881	0.00307	0.00000	0.00000
4	DRU	0.08249	0.00000	0.06008		0.72765	0.00149	0.20483	0.07752	0.11276	0.10217	0.00069	0.00000	0.00000
5	ZEI	0.08748	0.00000	0.01048	0.00000		0.00554	0.16266	0.07910	0.09672	0.10633	0.00129	0.00000	0.00000
6	ELE	0.08158	0.09350	0.25657	0.16011	0.21008		0.10890	0.19968	0.46778	0.11167	0.28096	0.02376	0.00000
7	KAU	0.06547	0.00000	0.12166	0.04188	0.05718	0.10883		0.14523	0.26057	0.13543	0.03099	0.00139	0.00000
8	ANT	0.03530	0.01867	0.11845	0.05446	0.08112	0.02641	0.07452		0.72389	0.63726	0.11504	0.00713	0.00000
9	NER	0.01300	0.00000	0.14183	0.04338	0.07464	0.00000	0.02946	0.00000		0.46926	0.11375	0.00950	0.00000
10	SMA	0.04631	0.04134	0.13518	0.07268	0.10464	0.07473	0.10675	0.00000	0.00000		0.11732	0.00653	0.00000
11	KUR	0.20552	0.36784	0.50305	0.44421	0.50951	0.08543	0.42405	0.13809	0.15418	0.19881		0.62647	0.13494
12	NEM	0.19941	0.26371	0.40781	0.32721	0.36444	0.08638	0.27946	0.13076	0.12717	0.20019	0.00000		0.00327
13	PLA	0.65336	0.76422	0.80085	0.67649	0.80585	0.47222	0.81567	0.52405	0.54380	0.66186	0.39012	0.15675	

Pairwise F_{ST} comparisons and the statistical significance of these values (P) are presented below and above the diagonal, respectively.

Table S4. Distribution of *R. rutilus* and *P. fluviatilis* haplotypes and network-defined clusters across sampling locations.

Location Code	Size	Sub-basin	Haplotypes (Repetitions)	Subclusters (Repetitions)	Major clusters (Repetitions)
R. rutilus					
ANT	18	Šventoji River	H_1(1), H_2(3), H_3(1), H_4(1), H_5(1), **H_6(7)**, H_7(1), H_8(1), H_9(1), H_10(1)	**A(8)**, A1(3), A2(5), B1(2)	**A(16)**, **B(2)**
DUK	6	Dysna River	**H_6(3)**, H_10(2), H_18(1)	**A(4)**, B1(2)	**A(4), B(2)**
DRU	18	Dysna River	H_5(1), **H_6(3)**, H_10(4), H_11(2), H_12(1), **H_13(2)**, **H_14(2)**, H_15(1), H_16(1), H_17(1)	**A(6)**, A1(1), A3(2), A4(2), B1(5), B3(2)	**A(11)**, **B(7)**
DVA	20	Neris River	H_2(6), **H_6(8)**, H_7(1), H_19(1), H_20(1), H_21(1), H_22(1), H_23(1)	**A(10)**, A2(7), A5(1), B1(1), B2(1)	**A(18)**, **B(2)**
ELE	6	Strėva River	**H_6(3)**, H_17(1), H_24(1), H_25(1)	**A(5)**, A3(1)	**A(6)**
KAU	11	Nemunas River	H_2(2), H_5(2), **H_6(4)**, H_9(1), H_26(1), H_27(1)	**A(4)**, A1(2), A2(3), C(1), C1(1)	**A(9)**, **C(2)**
KUR	15	Nemunas River	H_2(2), **H_6(10)**, H_28(1), **H_29(2)**	**A(11)**, A1(2), A2(2)	**A(15)**
MER	15	Merkys River	**H_6(6)**, H_10(1), H_16(1), H_17(2), H_18(1), H_30(1), H_31(1), H_32(1), H_33(1)	**A(10)**, A3(3), B1(2)	**A(13)**, **B(2)**
MUS	12	Mūša River	**H_6(8)**, H_10(1), H_11(1), H_19(1), H_34(1)	**A(9)**, A4(1), B1(2)	**A(10)**, **B(2)**
NER	8	Neris River	H_2(2), **H_6(5)**, H_11(1)	**A(5)**, A2(2), A4(1)	**A(8)**
NEM	20	Nemunas River	**H_6(15)**, H_11(2), H_16(3)	**A(15)**, A3(3), A4(2)	**A(20)**
NEV	19	Nevėžis River	**H_6(7)**, H_7(1), H_16(2), H_25(1), H_35(2), H_36(1), H_37(1), H_38(1), H_39(1), H_40(1), H_41(1)	**A(11)**, A3(3), A4(4), B3(1)	**A(18)**, **B(1)**
SMA	16	Dysna River	H_2(3), H_4(2), H_5(1), **H_6(2)**, H_7(1), H_8(1), H_10(1), H_42(1), H_43(1), H_44(1), H_45(1), H_46(1)	**A(4)**, A1(3), A2(4), **B(2)**, B1(3)	**A(11)**, **B(5)**
ZEI	5	Žeimena River	**H_6(5)**	**A(5)**	**A(5)**
ZEM	20	Žeimena River	**H_6(12)**, H_7(1), H_24(1), H_35(1), **H_47(3)**, H_48(1), H_49(1)	**A(19)**, A4(1)	**A(20)**
GenBank	7		**H_6(3)**, H_50(1), H_51(1), H_52(1), H_53(1)	**A(3)**, **A6(2)**, D(1), D1(1)	**A(5)**, **D(2)**
P. fluviatilis					
DOT	8	Nevėžis River	**HAO1(2)**, HA2O1(1), **HCC(1)**, **HCD1(2)**, **HDF(1)**, HDO1(1)	**B2(1)**, C(3), C1(1), **E(1)**, E2(2)	**A(3)**, **B(1)**, C(4)

SIE	9	Siesartis River	**HAA(3)**, HAA2(1), HA3B(1), **HAB(1)**, HAC(1), **HCC(1)**, HDH(1),	**A(4)**, **A1(2)**, B4(1), **E(1)**, G(1)	**A(8)**, **B(1)**
DYS	14	Dysna River	**HAA(3)**, HAA8(1), **HAB(7)**, HA7B6(1), HBC(1), HCC8(1)	**A(4)**, **A1(8)**, B(1), E2(1)	**A(13)**, **B(1)**
ZEI	12	Žeimena River	**HAA(3)**, **HAB(5)**, **HCC(3)**, HC8A(1)	**A(3)**, **A1(5)**, **E(4)**	**A(12)**
DRU	31	Dysna River	**HAA(6)**, HA4A(1), HAC(1), HA4C(1), **HAB(10)**, HAB1(1), HBC(1), HBJ(1), **HCC(8)**, HC2C(1)	**A(9)**, **A1(10)**, A3(1), B(2), C(9)	**A(20)**, **B(2)**, C(9)
NEM	30+2	Nemunas River	**HAA(2)**, **HAB(1)**, HBJ3(1), **HCC(3)**, HCJ(1), HC3C(1), HC3D(1), HC4C(1), **HDF(11)**, HDF6(1), HDF8(1), HD4F(1), HD5F(1), HD6F(1), HDH(3), HDL4(1), HDL6(1)	**A(2)**, **A1(1)**, B(1), B1(1), **B2(14)**, B3(1), B4(3), **E(6)**, E2(1)	**A(10)**, **B(20)**
NER	15	Neris River	**HAA(1)**, HAA6(1), HAT(2), **HAB(1)**, HBC(3), HCA(1), **HCC(3)**, HDT(1), **HDF(1)**, HD7F(1)	**A(2)**, **A1(1)**, A2(3), B(3), **B2(2)**, **E(4)**	**A(10)**, **B(5)**
KUR	3	Nemunas River	HDB(1), **HDF(2)**	A5(1), **B2(2)**	**A(1)**, **B(2)**
ELE	19	Strėva River	**HAA(3)**, **HAB(4)**, **HCC(2)**, **HDC(7)**, HD5C(1), **HDF(1)**, HD3F(1)	**A(3)**, **A1(4)**, B1(8), **B2(2)**, **E(2)**	**A(9)**, **B(10)**
SMA	8	Dysna River	**HAB(2)**, HAB8(1), HBJ(1), **HBJ6(2)**, **HCC(1)**, HCQ(1)	**A1(3)**, B(3), **E(1)**, E1(1)	**A(5)**, **B(3)**
ANT	16+2	Šventoji River	**HAA(1)**, **HAB(2)**, HAT(2), HAT1(1), **HBJ4(3)**, HBL10(1), HBL11(1), **HCC(4)**, **HDF(3)**	**A(1)**, **A1(2)**, A2(2), A4(1), B(3), **B2(3)**, **E(4)**	**A(10)**, **B(6)**
KAU	7	Nemunas River	**HAA(5)**, HAB1(1), **HDF(1)**	**A(5)**, A3(1), **B2(1)**	**A(6)**, **B(1)**
PLA	19	Minija River	**HDF(18)**, HDH(1)	**B2(18)**, B4(1)	**B(19)**
GenBank	8+2		HDE(1), **HDF(4)**, **HDL12(2)**, HFM(1), **HGC(2)**	**B2(4)**, B5(1), D(1), **F(2)**	**B(7)**, D(1)

R. rutilus haplotypes: H_6: OR263623.1 (Australia), OX637958.1 (United Kingdom), PP928771.1 (Czech Republic); H_50: OP612947.1 (China); H_51: OM736800.1 (Romania); H_52: NC_068671.1 (Romania); H_53: AP010775.1 (Russia). *P. fluviatilis* haplotypes: HDE: OP882669.1 (Australia); HDF: MT410943.1 (Denmark), CM020933.1 = VHII01000304.1 (France; same individual), PP059108.1 (Australia), OP882670.1 (Australia); HDL12: LC495488.1 (Denmark), AP018422.1 (Denmark); HFM: KM410088.1 = NC_026313.1 (Hungary; same individual); HGC: MZ461595.1 (China), AP005995.1 (China). A few haplotypes exhibiting heteroplasmic length variation are displayed separately by sample size and marked in green. Green-labelled haplotypes indicate singletons and doubletons (bold) containing heteroplasmic length variations with point mutations, respectively. Black bold indicates haplotypes and clusters occurring in more than 50% of studied locations per species. Red indicates haplotypes and clusters detected in a given location once (regular font) or more than once (bold red). Clusters are defined based on haplotype network topology and are used for descriptive purposes only; they do not represent phylogenetically supported lineages.

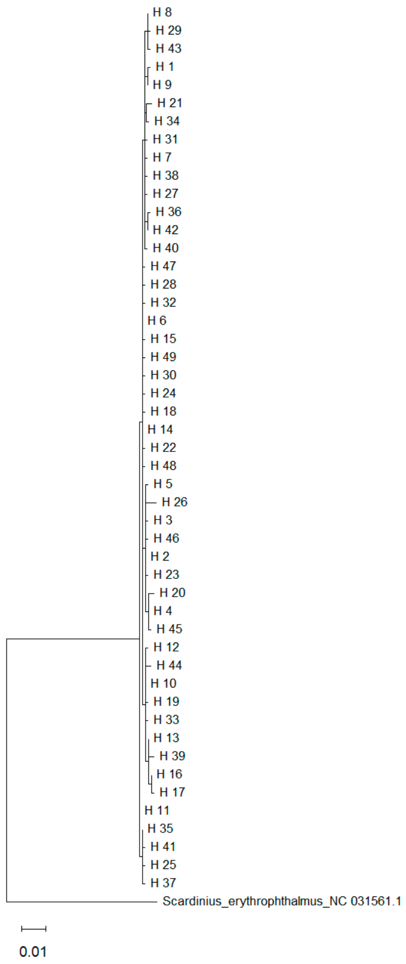

Figure S1. Maximum Likelihood phylogenetic tree of *R. rutilus* haplotypes inferred using the HKY+G nucleotide substitution model. The tree was rooted using *Scardinius erythrophthalmus* as an outgroup. Only bootstrap values ≥70% are shown.

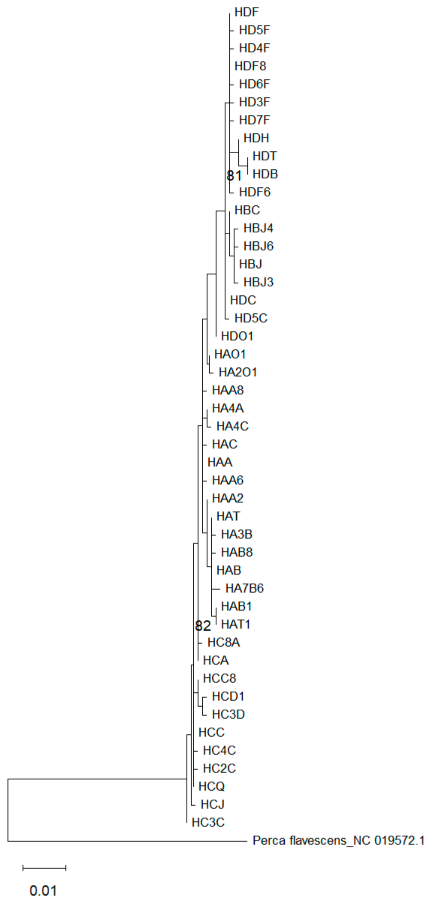

Figure S2. Maximum Likelihood phylogenetic tree of *P. fluviatilis* haplotypes inferred using the HKY+I nucleotide substitution model. The tree was rooted using *Perca flavescens* as an outgroup. Only bootstrap values ≥70% are shown.
